# The antischistosomal potential of GSK-J4, an H3K27 demethylase inhibitor: insights from molecular modeling, transcriptomics and *in vitro* assays

**DOI:** 10.1186/s13071-020-4000-z

**Published:** 2020-03-17

**Authors:** Jessica Lobo-Silva, Fernanda J. Cabral, Murilo S. Amaral, Patrícia A. Miyasato, Rafaela Paula de Freitas, Adriana S. A. Pereira, Mariana I. Khouri, Mayra M. F. Barbosa, Pablo I. P. Ramos, Luciana C. C. Leite, Oluwatoyin A. Asojo, Eliana Nakano, Sergio Verjovski-Almeida, Leonardo P. Farias

**Affiliations:** 1 Laboratório de Biomarcadores e Inflamação, Instituto Gonçalo Moniz, Fundação Oswaldo Cruz, Salvador, Bahia Brazil; 2 Departamento de Biologia Animal, Instituto de Biologia, Universidade Estadual de Campinas, Campinas, SP Brazil; 3 Laboratório de Expressão Gênica em Eucariotos, Instituto Butantan, São Paulo, SP Brazil; 4 Laboratório de Parasitologia, Instituto Butantan, São Paulo, SP Brazil; 5 Departamento de Bioquímica, Instituto de Química, Universidade de São Paulo, São Paulo, Brazil; 6 Laboratório Especial de Desenvolvimento de Vacinas, Instituto Butantan, São Paulo, SP Brazil; 7 Centro de Integração de Dados e Conhecimentos para Saúde (CIDACS), Instituto Gonçalo Moniz, Fundação Oswaldo Cruz, Salvador, Bahia Brazil; 8 Department of Chemistry and Biochemistry, Hampton University, Hampton, VA USA

**Keywords:** Anthelmintic drug discovery, Epigenetics, Jumonji histone demethylase

## Abstract

**Background:**

Schistosomiasis chemotherapy is largely based on praziquantel (PZQ). Although PZQ is very safe and tolerable, it does not prevent reinfection and emerging resistance is a primary concern. Recent studies have shown that the targeting of epigenetic machinery in *Schistosoma mansoni* may result in severe alterations in parasite development, leading to death. This new route for drug discovery in schistosomiasis has focused on classes of histone deacetylases (HDACs) and histone acetyltransferases (HATs) as epigenetic drug targets. *Schistosoma* histone demethylases also seem to be important in the transition of cercariae into schistosomula, as well as sexual differentiation in adult worms.

**Methods:**

The Target-Pathogen database and molecular docking assays were used to prioritize the druggability of *S. mansoni* histone demethylases. The transcription profile of Smp_03400 was re-analyzed using available databases. The effect of GSK-J4 inhibitor in schistosomula and adult worms’ motility/viability/oviposition was assessed by *in vitro* assays. Ultrastructural analysis was performed on adult worms exposed to GSK-J4 by scanning electron microscopy, while internal structures and muscle fiber integrity was investigated by confocal microscopy after Langeronʼs carmine or phalloidin staining.

**Results:**

The present evaluation of the potential druggability of 14 annotated *S. mansoni* demethylase enzymes identified the *S. mansoni* ortholog of human KDM6A/UTX (Smp_034000) as the most suitable druggable target. *In silico* analysis and molecular modeling indicated the potential for cofactor displacement by the chemical probe GSK-J4. Our re-analysis of transcriptomic data revealed that Smp_034000 expression peaks at 24 h in newly transformed schistosomula and 5-week-old adult worms. Moreover, this gene was highly expressed in the testes of mature male worms compared to the rest of the parasite body. In *in vitro* schistosome cultures, treatment with GSK-J4 produced striking effects on schistosomula mortality and adult worm motility and mortality, as well as egg oviposition, in a dose- and time-dependent manner. Unexpectedly, western blot assays did not demonstrate overall modulation of H3K27me3 levels in response to GSK-J4. Confocal and scanning electron microscopy revealed the loss of original features in muscle fibers and alterations in cell-cell contact following GSK-J4 treatment.

**Conclusions:**

GSK-J4 presents promising potential for antischistosomal control; however, the underlying mechanisms warrant further investigation.
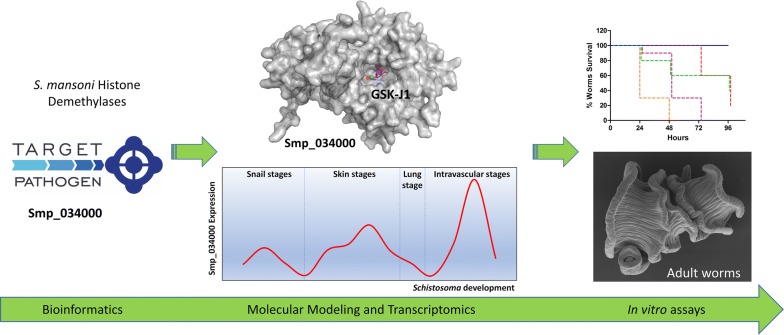

## Background

Schistosomiasis, a chronic disease caused by trematodes of the genus *Schistosoma*, affects more than 230 million people living in tropical and subtropical areas, resulting in up to 300,000 deaths annually [1]. This neglected tropical disease (NTD), which is responsible for up to 1.9 million disability-adjusted life years (DALYs) lost annually, remains one of the most relevant to date [2].

Due to the lack of an available vaccine, mass drug administration (MDA) of praziquantel (PZQ), a safe and potent chemotherapy developed in the mid-1970s, continues to be the main control strategy in use; however, mass treatment does not prevent reinfection and its cumulative effects [3]. Furthermore, the emergence of drug-resistant parasites is of concern [4], highlighting the need to search for new drug targets.

The *S. mansoni* transcriptome [5] and genome [6] projects have paved the way toward the identification of potential drug targets, as targeting specific gene products or pathways can be envisaged *via in silico* analysis. After mining pertinent pathways, a “piggy-backing” strategy can be applied to focus on drug targets already validated in other human diseases for which chemical probes are available. Furthermore, this approach offers potential timesaving and cost benefits in the context of NTDs, which face investment constraints in relation to drug discovery. Initially, a chemogenomic screening pipeline pinpointed some schistosome proteins upon which existing drugs may act against, including classes of lipid metabolism, G protein-coupled receptors (GPCRs), ligand- and voltage-gated ion channels, kinases, proteases and neuropeptides [6, 7], of which some have been validated [8, 9].

In addition, the complexity of the schistosome life-cycle, i.e. different intra-molluscan, aquatic-dwelling and intra-vertebrate stages, requires highly controlled gene transcription, suggesting that epigenetic mechanisms are likely involved in parasite development and differentiation [10]. This complex regulation is achieved *via* the action of: (i) non-protein-coding RNAs (ncRNAs) [11, 12]; (ii) histone *writer* enzymes, which add epigenetic marks (e.g. histone acetyltransferases (HATs) and methyltransferases (HMTs)); (iii) *readers,* which read epigenetic marks (e.g. bromodomains, chromodomains and PHD fingers-containing proteins); and (iv) *eraser* enzymes, which remove epigenetic marks (e.g. histone deacetylases (HDACs) and demethylases (HDMs)) [13]. Importantly, experimental evidence suggests that targeting key epigenetic players could represent a promising approach for drug discovery against eukaryotic pathogens (reviewed by [14, 15]).

Over the last decade, several HDAC/HAT inhibitors originally designed for cancer treatment have been investigated in the context of schistosomiasis. The HDAC inhibitor Trichostatin A (TSA) was described as blocking the *in vitro* transformation of *S. mansoni* miracidia into primary sporocysts [16], and also found to ablate strain-specific differences in the expression of miracidia parasites [16–18]. In addition, this inhibitor successfully induced the death of adult worms and schistosomula in culture [17]. Subsequently, similar effects have been observed using sirtuins, a class III HDAC inhibitor [19]. Marek et al. [20] used a structure-based approach to identify several inhibitors of the *S. mansoni* HDAC8 enzyme capable of inducing apoptosis and parasite mortality, which also presented low affinity to its human counterpart. The HAT PU139 inhibitor was also found to arrest egg development by hyperacetylation of histone H3 at lysine 9 (H3K9ac) [21].

Histone demethylases have also come under increasing scrutiny as drug targets for anticancer therapy [22]. Recently, Roquis et al. [23] provided evidence that trimethylation of histone H3 at lysine 27 (H3K27me3) is removed during cercariae transformation into schistosomula and remains absent in adult worms. Furthermore, these authors described changes in H3K9 methylation and acetylation both upstream and downstream of the transcriptional start site [23]. This data highlights the importance of HMTs and HDMs for gene regulation and parasite development. Two families of proteins exert demethylase activity, the K-demethylases (KDMs) or lysine specific demethylase (LSD), and the Jumonji C (JMJC) domain-containing demethylases [24]. In humans, the demethylases are comprised of 24 family members (https://www.genenames.org/cgi-bin/genefamilies/set/485), while in the schistosome, 14 putative histone demethylase (HDM) enzymes were recently described [25].

In an attempt to explore *S. mansoni* histone demethylases (SmHDM) as putative drug targets, we combined *in silico* and *in vitro* approaches. The druggable potential of this class of enzymes was initially assessed using computational tools, disclosing *S. mansoni* Smp_034000 as a potential target. We then used molecular modeling to predict site-specific interactions between Smp_034000 and the chemical probe GSK-J4, an inhibitor of mammalian KDM6A (UTX) and KDM6B (JMJD3) developed by [26]. Finally, the chemotherapeutic potential of GSK-J4 was evaluated in adult worms and schistosomula *in vitro* cultures; GSK-J4 was found to induce parasite mortality, with an IC_50_ close to that established of inhibiting the mammalian enzyme. Although our *in silico* and *in vitro* approaches suggest that GSK-J4 represents a promising *Schistosoma* KDM6-demethylase inhibitor, the exact molecular mechanism(s) underlying the observed effects remain(s) unelucidated. The present findings are discussed in light of recently published findings by another group that evaluated similar compounds [27].

## Methods

### Parasites

*Schistosoma mansoni* (BH strain) was maintained at the Laboratory of Parasitology of Butantan Institute. Cercariae were acquired *via* the exposure of infected *Biomphalaria glabrata* snails to bright light. Schistosomula were obtained by the mechanical transformation of cercariae as previously described [28], followed by *in vitro* cultivation for 3 h using a modified protocol [29]. Hamsters were infected with 300 cercariae, and 6–7 weeks after infection, adult worms were recovered by portal perfusion using RPMI-1640 medium buffered with 10 mM HEPES (Invitrogen, Paisley, UK) and 500 units/l of heparin.

### *In silico* analysis of *S. mansoni* demethylation enzymes

To evaluate druggability and prioritize the *S. mansoni* histone demethylases, the Target-Pathogen database and the recently described data available in Padalino et al. [25] were used. The Target-Pathogen tool allows for genome-scale target prioritization based on structural protein data, omics experiments, essentiality and metabolic context [30]. Particularly, structural druggability data for SmHDM were retrieved, which are calculated based on prediction of cavities in the tridimensional structure of the protein and the evaluation of its target potential using the *fpocket* tool [31]. The Simple Modular Architecture Research Tool (SMART) (http://smart.embl-heidelberg.de/) was used for domain predictions [32]. Further analysis of the sequence in the catalytic domain of the Smp_034000 protein in relation to human UTX/KDM6A (UniProt: O15550), JmjD3/KDM6B (UniProt: O15054), and UTY (UniProt: O14607), was done through alignment performed by ClustalX and visualization by Genedoc.

### Transcription profiling

After SmHDM druggability analysis, expression data from the 37,632 elements contained in the *S. mansoni* oligonucleotide DNA microarray reported by Fitzpatrick et al. [33] was interrogated to determine the expression profile of Smp_034000 across 15 different life-cycle stages. Raw and normalized fluorescent intensity values available *via* Array Express (https://www.ebi.ac.uk/arrayexpress/) under experiment accession number E-MEXP-2094, were retrieved. A re-annotation of the microarray probes was performed using version 5.2 of the predicted *S. mansoni* mRNAs (fasta) and corresponding gff files downloaded from GeneDB, as the original annotation from the Fitzpatrick et al. [33] study was based on version 4. BLASTN of all 50-mer probes was run against the annotated GeneDB *S. mansoni* models using the “*task blastn-short*” parameter, which is appropriate for short sequences. The identity and coverage of the hits were recorded, and the alignments for each gene of interest were visually inspected. Expression data was presented as the average signal of validated probes. RNA-seq data on the expression of Smp_034000 was extracted from a previously published dataset [34] available *via* the European Nucleotide Archive repository, (https://www.ebi.ac.uk/ena/browser/home) under experiment accession number PRJEB14695, presenting gonad-specific and pairing-dependent transcriptomes, for which only |log2 fold-change| ≥ 1.0 (FDR < 0.05) were considered.

### Inhibitor docking

Amino acid residues corresponding to the JmjC domain of the Smp_034000 protein were submitted for automatic homology modeling for protein structure prediction using Phyre2 (http://www.sbg.bio.ic.ac.uk/phyre2/html/page.cgi?id=index). The two structures of the human homologue that contain a ligand of interest, with coordinates deposited in the protein databank have PDB codes 2XUE and 2XXZ were used. The atomic coordinates for these structures were downloaded and used in docking and modeling studies. The model generated from Phyre2 was superimposed with each of the coordinates of the human homologue (2XUE and 2XXZ) using the least squares alignment function of Pymol (The PyMOL Molecular Graphics System, Version 1.2r3pre, Schrödinger, LLC). The coordinates corresponding to the JmjC domain and each ligand were extracted, combined, and exported into a single file. The resulting model containing a ligand and the JmjC domain of the Smp_034000 was submitted for structure optimization and idealization using REFMAC5 refinement program [35] of the CCP4 package [36]. The ligand-protein interactions for each model were visualized and compared using Ligplot [37].

### Inhibitors

Initially, the chemical probe GSK-J4 and its inactive isomer (GSK-J5) were kindly donated by Dr Susanne M. Knapp (Oxford University and SCG consortium) and subsequently purchased from Sigma-Aldrich (St. Louis, USA) and Tocris (Bristol, UK), respectively. PZQ was purchased from Sigma-Aldrich.

### Schistosome *in vitro* cultures and GSK-J4 treatment

For adult worm drug treatment, paired mature couples of adult worms (male and female) were washed in RPMI-1640 after perfusion. For each treatment condition, 10 worm pairs were incubated on 24-well culture plates at 37 °C under 5% CO_2_; each well contained one pair of worms in 1 ml of RPMI-1640 supplemented with 10% fetal bovine serum (FBS), 100 μg/ml penicillin/ streptomycin and 0.5 μg/ml amphotericin B (Sigma-Aldrich) and differing concentrations of either GSK-J4 (1, 5, 10, 20 and 30 μM), GSK-J5 (30 μM), PZQ (4.8 µM) or 1% dimethyl sulfoxide (DMSO). After 24, 48, 72 and 96 h, treated and control worms were evaluated by stereo microscopy and scored according to coupling (coupled or separated) and motility (normal, slightly reduced, significantly reduced or absent) as previously described [38]. Scoring data was expressed numerically to generate a heatmap. Worms lacking motility were considered dead and recorded by survival curves (Kaplan-Meier). All independent experiments were performed three times. An additional independent assay was performed to evaluate the effect of GSK-J4 and the inactive isomer GSK-J5 at lower concentrations of 200 nM, 800 nM, 1 µM, 5 µM and 20 µM after 72 h of treatment. Oviposition and total area dimension of *S. mansoni* eggs were assessed using ImageJ software (http://rsb.info.nih.gov/ij) following the acquisition of images using a light microscope coupled to a camera (Leica DMi8; Leica, Illinois, USA).

Evaluation of schistosomula after drug treatment was performed as previously described [39, 40] with modifications. Briefly, newly transformed schistosomula were maintained for 3 h in M169 (Vitrocell, Campinas, Brazil) medium supplemented with 2% FBS (Vitrocell), 1 μM serotonin, 0.5 μM hypoxanthine, 1 μM hydrocortisone, 0.2 μM triiodothyronine, penicillin/streptomycin, amphotericin and gentamicin (Vitrocell) at 37 °C under 5% CO2. GSK-J4 treatment was initiated after 3 h incubation. The viability of schistosomula was determined using the CellTiter-Glo Luminescent Cell Viability Assay (G7570; Promega, Madison, Wisconsin, EUA) after 24, 48, 72, 96 and 120 h of drug treatment. This assay quantifies the amount of ATP present in intact schistosomula and signals the presence of metabolically active cells.

### Electron microscopy analysis

Ultrastructural analysis was performed by scanning electron microscopy. Adult worms incubated in 20 μM GSK-J4 or the DMSO vehicle for 24 h were fixed in modified Karnovsky reagent (1% paraformaldehyde, 2.5% glutaraldehyde, 1 mM calcium chloride in 1M sodium cacodylate buffer, pH 7.4). After fixing, worms were washed with sodium cacodylate buffer (0.1 mol/l, pH 7.2) and post-fixed with 1% osmium tetroxide (w/v) for 1 h. The samples were then dehydrated with increasing concentrations of ethanol and dried with liquid CO_2_ using a critical-point dryer machine (Leica EM CPD030; Leica Microsystems, Illinois, USA). Treated specimens were mounted on aluminum microscopy stubs and coated with gold particles using an ion-sputtering apparatus (Leica EM SCD050; Leica Microsystems) [41]. Specimens were then observed and photographed using an electron microscope (FEI Quanta 250; Thermo Fisher Scientific, Oregon, USA).

### Langeron’s carmine and phalloidin staining

Adult worms were treated with 5 µM and 20 µM GSK-J5 or 800 nM, 7.5 µM and 20 µM GSK-J4 for 24 h. Treated worms were fixed overnight in AFA (2% acetic acid, 3% formaldehyde and 95% of 70% alcohol) at room temperature. Next, worms were stained overnight with alcoholic hydrochloric acid-Carmine (V001147; Vetec, Rio de Janeiro, Brazil) and then washed twice for 5 min with 0.5% hydrochloric acid in 80% ethanol. The worms were dehydrated in solutions containing increasing concentrations of ethanol 80, 90 and 100% at room temperature. Finally, xylol was used as a clarifying agent and the worms were mounted in Canada balsam mounting medium. Optical slices were obtained from whole-mount worms under a confocal laser scanning microscope (SP8, Leica) in differential interference contrast (DIC) and/or reflected mode, with excitation at 552 nm and a hybrid detector (569–703 nm).

Adult worms were also fixed using 4% paraformaldehyde in PBS, pH 7.4, for 4 h at room temperature, followed by two washes in PBS and storage at 4 °C until use. The specimens were permeabilized in permeabilizing buffer solution (PBS, 1% Triton X-100, 0.1% SDS) and then incubated for 16 h at 4 °C in a solution (PBS, 0.3% Triton X-100, 0.05% Tween-20) containing phalloidin-fluorescein isothyocianate (FITC) (0.25 µg/ml) for musculature staining and 4′,6-diamidin-2-phenylindol (DAPI) (1 µg/ml) for nuclei staining. Next, parasites were washed three times in PBS at 4 °C, mounted on slides using ProLong Gold Antifade Mountant (Thermo Fisher Scientific) and confocal microscopy using the same equipment was performed. Images were captured at a wavelength of 488 nm for FITC and 358 nm for DAPI.

### Acid histone extraction and western blotting

After 24 h of treatment with 7.5 or 20 µM GSK-J4, histones were extracted from adult worms as previously described [17]. Briefly, adult worms (10 female or 10 male) were gently homogenized using a Dounce homogenizer in 1 ml PBS, 0.5% Triton X-100, 0.02% NaN_3_ and a protease inhibitor cocktail (P2714; Sigma-Aldrich). Cells were lysed on ice for 10 min under gentle stirring, and nuclei were isolated by centrifugation at 6500× *g* for 10 min at 4 °C, then washed in 0.5 ml of the same buffer and centrifuged again. For acid histone extraction, the nuclei pellet was sonicated (40–60 Hz for 1 min in an ice-bath) and resuspended in 400 µl of 0.25M HCl and incubated for 4 h under rocking agitation at room temperature. The samples were then centrifuged at 6500× *g* for 10 min at 4 °C to pellet debris. The supernatant containing the histone proteins was TCA-precipitated overnight and the pellet was washed twice with ice-cold acetone and resuspended in water with protease inhibitors and stored at − 20 °C until the time of western blot analysis.

After 72 h of treatment with 7.5 μM GSK-J4, nuclear proteins were extracted from schistosomula as previously described [40]. Briefly, 10,000 schistosomula were homogenized using a Dounce homogenizer in the same lysis buffer described above. Samples were then sonicated in an ultrasonic ice bath for 5 min (40 Hz) and centrifuged for 20 min at 13,000× *g* at 4 °C. The supernatant containing nuclear protein extract was separated and used for western blotting analysis.

Protein concentrations were determined by Bradford’s method (Protein Assay; Bio-Rad, Hercules, USA) and western blots were performed as previously described [42]. Briefly, 7.5 µg of adult and schistosomula histones were separated on 15% SDS-PAGE polyacrylamide gels and transferred to PVDF membranes, which were then incubated with 1:1000 anti-H3K27me3 (mAbcam 6002; Abcam, Cambridge, UK) and anti-H3 (mAbcam 24834; Abcam) monoclonal antibodies. Images were captured using an Image Quant LAS 4000 photo documentation system (GE Healthcare, Uppsala, Sweden). Image bands were measured by densitometric analysis using ImageJ software (http://rsweb.nih.gov/ij/).

### Statistical analysis

All data sets were tested for normality using D’Agostino-Pearson tests. Differences in fluorescence values for Smp_034000 (expression data) were analysed using a one-way ANOVA followed by Tukey’s multiple *post-hoc* comparison test. Drug effects on adult worms were recorded by Kaplan-Meier survival curves and *P*-values calculated using the log-rank (Mantel-Cox) test. The IC_50_ determination for schistosomula was performed using non-linear least squares curve fitting. Differences in oviposition were analyzed using the Kruskal-Wallis test followed by Dunn’s multiple comparison test, and differences in eggs area were analyzed using a one-way ANOVA followed by Tukey’s multiple *post-hoc* comparison test. Differences were considered statistically significant when *P* ≤ 0.05. All figures, Kaplan-Meier survival curves and statistical analyses were performed using PRISM version 5.02 software (GraphPad, San Diego, CA).

## Results

### Smp_034000 presents high druggability

The target-pathogen tool [30] was used to assess druggability of the 14 genome-annotated *S. mansoni* histone demethylases. The KDM6 (Smp_34000) ortholog was found to present the highest druggability score, thereby representing a potential target for the GSK-J4 chemical probe (Table 1).

**Table 1:** *Schistosoma mansoni* histone demethylase enzymes, corresponding accessions, human orthologs comparison, structural druggability and reported inhibitors

Wormbase ID^a^ size (aa) GeneDB ID^b^ size (aa)	Human ortholog (% ID) UniProt ID	Structural druggability^c^	Epigenetic inhibitor/ chemical probe^d^
Smp_150560^a^ (1164)	LSD1 (36%) O60341	0.752	GSK2879552, GSK-LSD1 [51] Tranylcypromine [52]
Smp_160810^a^ (916)	LSD2 (28%) Q8NB78	0.589	–
Smp_162940^a^ (1105)	LSD2 (27%) Q8NB78	0.543	–
Smp_161400^a^ (2575)	KDM3B (46%) Q7LBC6	0.766	JDI‐4 [53]
Smp_132170^a^ (984)	KDM4C (53%) Q9H3R0	0.718	4-hydroxypyrazole [54]
Smp_019170^a^ (1639)	KDM5D (41%) Q9BY66	0.891	–
Smp_156290^a^ (2612)	KDM5B (47%) Q9UGL1	0.848	KDOAM-25 [55]
Smp_034000^a^ (1393)	KDM6A (52%) O15550	0.921	GSK-J4 [26]
Smp_315890^a^ (483) Smp_127230^b, e^	KDM7A (39%) Q6ZMT4	0.84^e^	Daminozide (N-(dimethylamino) succinamic acid [56]
Smp_213920^a^ (401) Smp_147870^b, e^	JMJD4 (40%) Q9H9V9	0.801^e^	–
Smp_316180^a^ (782)	JMJD6 (61%) Q6NYC1	0.857	–
Smp_342360^a^ (353) Smp_128500^b, e^	JMJD5 (41%) Q8N371	0.688^e^	–
Smp_241580^a^ (396) Smp_180990^b, e^	JMJD5 (25%) Q8N371	0.854^e^	–
Smp_333400^a^ (771) Smp_173670^b, e^	NO66/RIOX1 (26%) Q9H6W3	0.764^e^	–

^a^*Schistosoma mansoni* Wormbase ID (genome assembly version 7.0)

^b^*Schistosoma mansoni* GeneDB ID (genome assembly version 5.2)

^c^Druggability prediction with target-pathogen [30]

^d^Inhibitors described in [26, 51–56]

^e^Data obtained using contigs, containing UniProt database ID, from previous *Schistosoma* genome assembly (v. 5.2)

With respect to the overall domain architecture of proteins with high druggability scores and presenting inhibitors already described, Smp_034000 appears to be the most conserved in relation to the human enzymes. Smp_034000 is more closely related to human KDM6A/UTX than to human KDM6B/JmJD3, with the JmjC and tetratricopeptide repeat (TPR) domains spanning similar regions in the schistosome and mammalian proteins (Additional file 1: Figure S1).

### Smp_034000 expression peaks at 24 h after cercarial transformation and at 5 weeks in adult worms

In an attempt to acquire insight regarding the regulation of Smp_034000 across fifteen parasite life-cycle stages (including intra-molluscan, aquatic-dwelling and intra-vertebrate stages), we interrogated data from the 37,632 element *S. mansoni* DNA microarray study published by Fitzpatrick et al. [33] to recover the expression profile of Smp_034000. It is interesting to note the peak of expression at 5-week adult worm stage (ANOVA: *F*_(14, 30)_ = 4.336, *P* = 0.0004). Moreover, higher levels were observed in 24 h schistosomula, although not statistically significant (Additional file 2: Figure S2a). Regarding comparisons of gonad-specific and pairing-dependent gene expression (i.e. single-sex infections *versus* bi-sex infection) performed by Lu et al. [34], Smp_034000 KDM6A expression in the testes was two-fold higher than the rest of the parasite body in male worms in both single-sex male infection and bi-sex infection models (Additional file 2: Figure S2b). In relation to the expression of this gene in females, two-fold higher expression was observed in the ovaries *versus* the rest of the female parasite body only in a model of single-sex female worm infection, i.e. not in the bi-sex infection model (Additional file 2: Figure S2b).

### Interaction between GSK-J4 and the active site

Concerning the primary structure of Smp_034000, analysis of the alignment of the catalytic domain in relation to human KDM6A and UTY revealed the conservation of almost all residues participating in enzyme activity (Additional file 3: Figure S3). We can highlight the residues involved in histone binding (red stars), residues participating in cofactor binding (NOG) to metal binding (blue circles), and finally the cysteines that participate in Zinc binding (green triangles) (Additional file 3: Figure S3). The only exception found was the substitution of a Ser by an Ala (position ~1460) at the metal binding site (dark blue circle). This changes the charge of the residue and it is unknown what the significance of this modification is on function. In addition, the alignment of *S. mansoni* enzymes with human orthologs revealed additional stretches of 25, 14 and 20 amino acids of unknown function in the schistosome protein structure. Structural comparisons suggest that the insertions are in surface loops, which may not directly influence the catalytic activity. Due to the difficulty in modeling loop regions, it is possible that these loops may play a greater role than suggested by the homology models so future studies will include generating crystal structures to clarify what roles these extended loops may play in the presence and absence of the inhibitors.

**Fig. 1:**
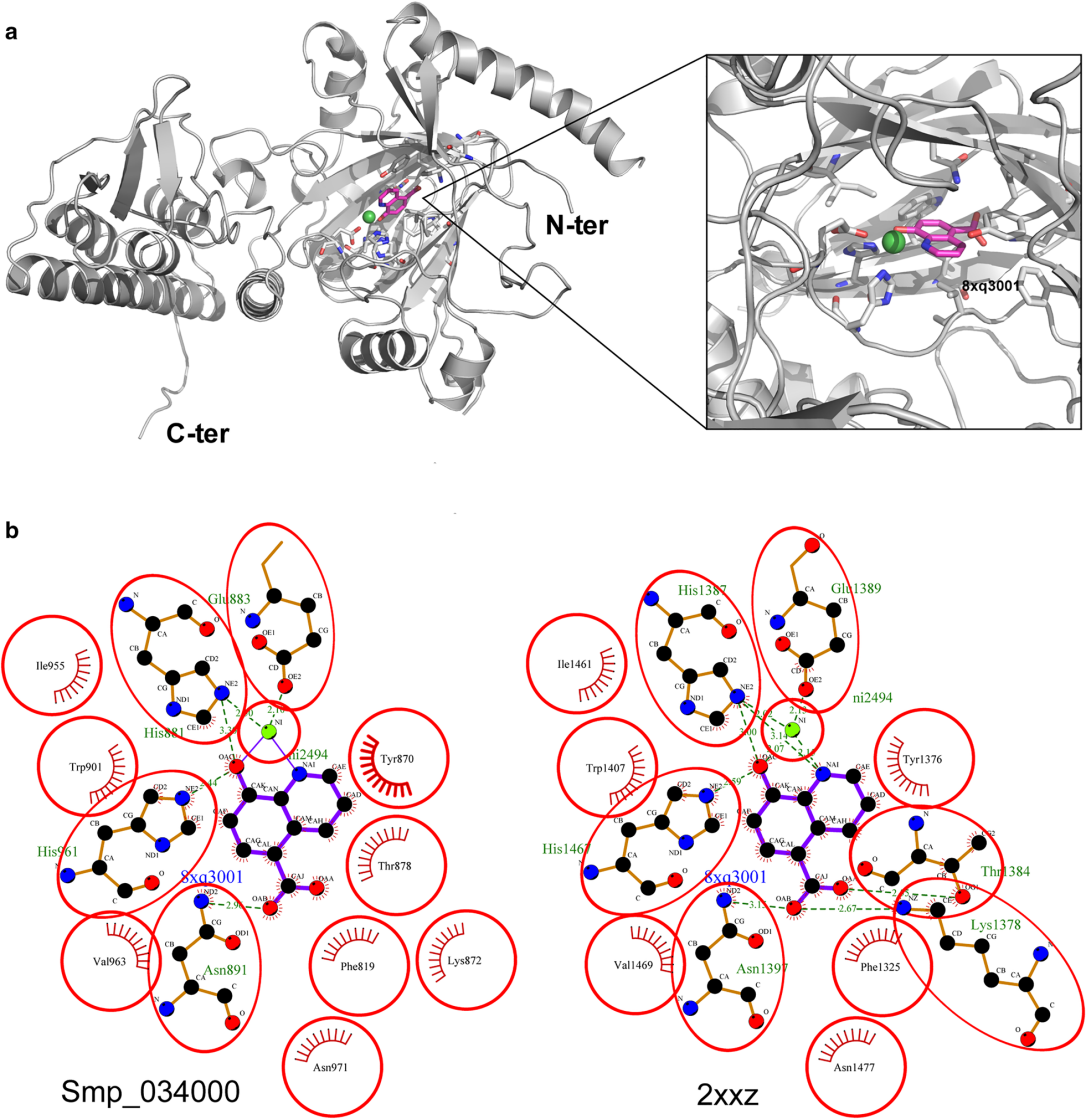
Three-dimensional modeling of Smp_034000 protein and GSK-J4 inhibitor. **a** Homology model of target protein with ligand 8xq3001 and Ni ion in cavity. The inset shows the cavity of target protein with inhibitor 8xq3001 (magenta) and Ni2+ ion (green), the interacting amino acid residues are shown in stick. **b** Predicted interactions of GSK-J1 with Smp_034000 putative Nickel binding site. The binding sites of Nickel atom in the structure of crystal structure of human JMJD3 (PDB code 2xxz) is presented for comparison (prepared by LIGPLOT)

Three-dimensional modeling of Smp_034000, based on the human KDM6A protein and its interaction with the GSK-J1 inhibitor (the active form of GSK-J4 after cell penetration), suggests that the inhibitor may interact with the active site of the Smp_034000 enzyme in the presence of a Ni^+2^ ion (Fig. 1a). Comparisons between the human protein cavity with the GSK-J1 inhibitor and the Smp_034000 cavity reveals a high conservation of amino acids essential to inhibitor interaction, notably Glu 883, His 881, His 961 and Asn 891 (Fig. 1b).

Based on our expression data analysis, the druggability score, and the important role that the H3K27me3 mark could play in *S. mansoni* development, we endeavored to investigate the potential of the Smp_034000 enzyme as drug target against *S. mansoni.*

### GSK-J4 inhibitor induces *in vitro* mortality in adult worms and schistosomula

**Fig. 2:**
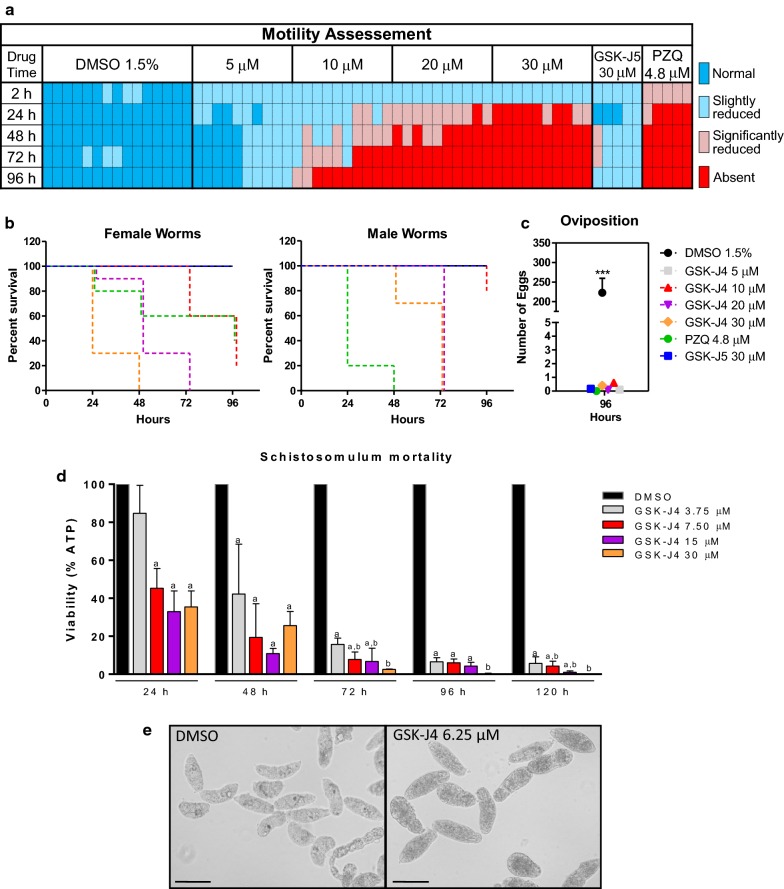
*In vitro* effects of GSK-J4 on the motility and viability of *S. mansoni* adult worms and schistosomulum. **a** Adult worms were treated with different concentrations of GSK-J4, PZQ, GSK-J5 or DMSO alone and motility phenotypes were scored at different time points as described in Methods, each cell represents a couple of worms. **b** Kaplan-Meier survival curves of adult female and male schistosomes cultured for 4 days following treatment with different concentrations of GSK-J4 and controls. **c** Number of eggs released by couples of worms treated with GSK-J4 and controls, ***P < 0.001. Data is representative of one from three independent experiments. **d** Schistosomula (100 per well) were incubated in quadruplicate with serial dilutions of GSK-J4 or with vehicle (0.1% DMSO) for up to 5 days. The viability was expressed as percentage of the luminescence values relative to the control (DMSO). **e** Microscopic assessment of schistosomula treated with 6.25 μM GSK-J4 for 48 h revealing a granulation phenotype. Mean ± SD from three replicate experiments. Letters indicate significant differences between treatments (*P* < 0.05) (one-way ANOVA). *Scale-bars*: 100 µm

In order to determine the anti-schistosomal potential of GSK-J4, cultured adult worms and schistosomula were exposed to different concentrations of the compound. Adult worm viability was assessed by daily optical examinations and scored according to coupling and motility, which were then converted to numerical scores to generate a heatmap. The compound was observed to impair motility and induce mortality in adult worms in a time- and concentration-dependent manner (Fig. 2a, b). A slight reduction in motility was observed after 24 h of treatment with 5.0 μM GSK-J4, which became more pronounced at higher concentrations (10–20 μM). Treatment with 30 μM of the inactive isomer (GSK-J5) promoted minor alterations in worm motility (Fig. 2a).

Regarding mortality, the first dead females were seen at 24 h of treatment with 20 μM GSK-J4 (Fig. 2b), which progressively increased in rate, reaching 70% and 100% at 48 h and 72 h, respectively. Although female worms seemed to be more sensitive than males, both reached a 100% death rate at 72 h under 20 μM GSK-J4. PZQ was used as a positive assay control and was confirmed to be less effective in female worms [43]. A decrease in oviposition was already observed with the lowest concentrations of GSK-J4 (5 μM). Although not inducing mortality, treatment with 30 μM GSK-J5 (the inactive isomer) inhibited female oviposition similarly to GSK-J4 (ANOVA: *F*_(6, 57)_ = 22.90, *P* < 0.0001) (Fig. 2c). To further investigate this effect, couples of adult worms were treated for 72 h with low concentrations of GSK-J4 (200 nM, 800 nM, 1 μM and 5 μM) and GSK-J5 (200 nM, 800 nM, 1 μM, 5 μM and 20 μM). Our results indicated a pronounced reduction (~97%) in oviposition after treatment with 200 nM of GSK-J4 (Kruskal-Wallis, *H*_(4)_ =15.06, *P* = 0.0046) (Additional file 4: Figure S4a). This effect was only observed with 30 μM of GSK-J5 with ~99% inhibition in oviposition (Fig. 2c, Additional file 4: Figure S4a) (Kruskal-Wallis, *H*_(6)_ =12.57, *P* = 0.0504). Noteworthy, the eggs derived from 800 nM GSK-J4 treatment were phenotypically different from control eggs, displaying short spines, shape alterations and ~66% reduction in area (ANOVA: *F*_(7, 146)_ = 38.31, *P* < 0.0001) (Additional file 4: Figure S4b, c). A slight decrease in egg area (~18%) was also observed after 200 nM of GSK-J4 exposure, but without any other alteration (e.g. shape, shell or spine alterations). Unexpectedly, the inactive isomer (GSK-J5) showed a slightly reduction of egg size (~20%) at 20 μM (ANOVA: *F*_(7, 146)_ = 38.31, *P* < 0.0001), but with no other significant alteration (Additional file 4: Figure S4b, c).

Next, to further investigate the potential of GSK-J4, we evaluated its schistosomicidal effect in 3 h newly transformed schistosomula by determining the decrease in viability according to the percentage of ATP reduction in the parasites after GSK-J4 treatment. Concentrations ranging between 3.75–30.0 μM were added to *in vitro* parasite cultures (100 schistosomula/well) and assayed after 24, 48, 72, 96 and 120 h. Our results indicated that schistosomula viability was significantly impaired as early as 24 h. For 7.5 μM, schistosomula viability decreased to 54%, 80%, 92% and 95% at 24 h (ANOVA: *F*_(4, 15)_ = 32.84, *P* < 0.0001), 48 h (ANOVA: *F*_(4, 15)_ = 23.93, *P* < 0.0001), 72 h (ANOVA: *F*_(4, 11)_ = 463.1, *P* < 0.0001) and 120 h (ANOVA: *F*_(4, 11)_ = 2008, *P* < 0.0001), respectively. A slightly higher effect was observed at higher concentrations, as at 15 μM schistosomula viability decreased to 67%, 89%, 93% and 98% at 24 h, 48 h, 72 h and 120 h, respectively (Fig. 2d). The half maximal-inhibitory concentration (IC_50_) of GSK-J4 was determined to be 4.2 μM. The respective selective-index (SI) was found to be either > 6.0 based on the total amount of ATP available in treated HEK293 cells using a luminescent viability assay, or > 15.0 based on an Alamar Blue cytotoxicity assay involving bone marrow-derived macrophages. Images and videos illustrate the effect of the compound at a concentration of 6.25 μM GSK-J4 after 48 h of treatment (Fig. 2e) and (Additional file 5: Movie S1).

### H3K27me3 histone mark levels in adult worms and schistosomula

**Fig. 3:**
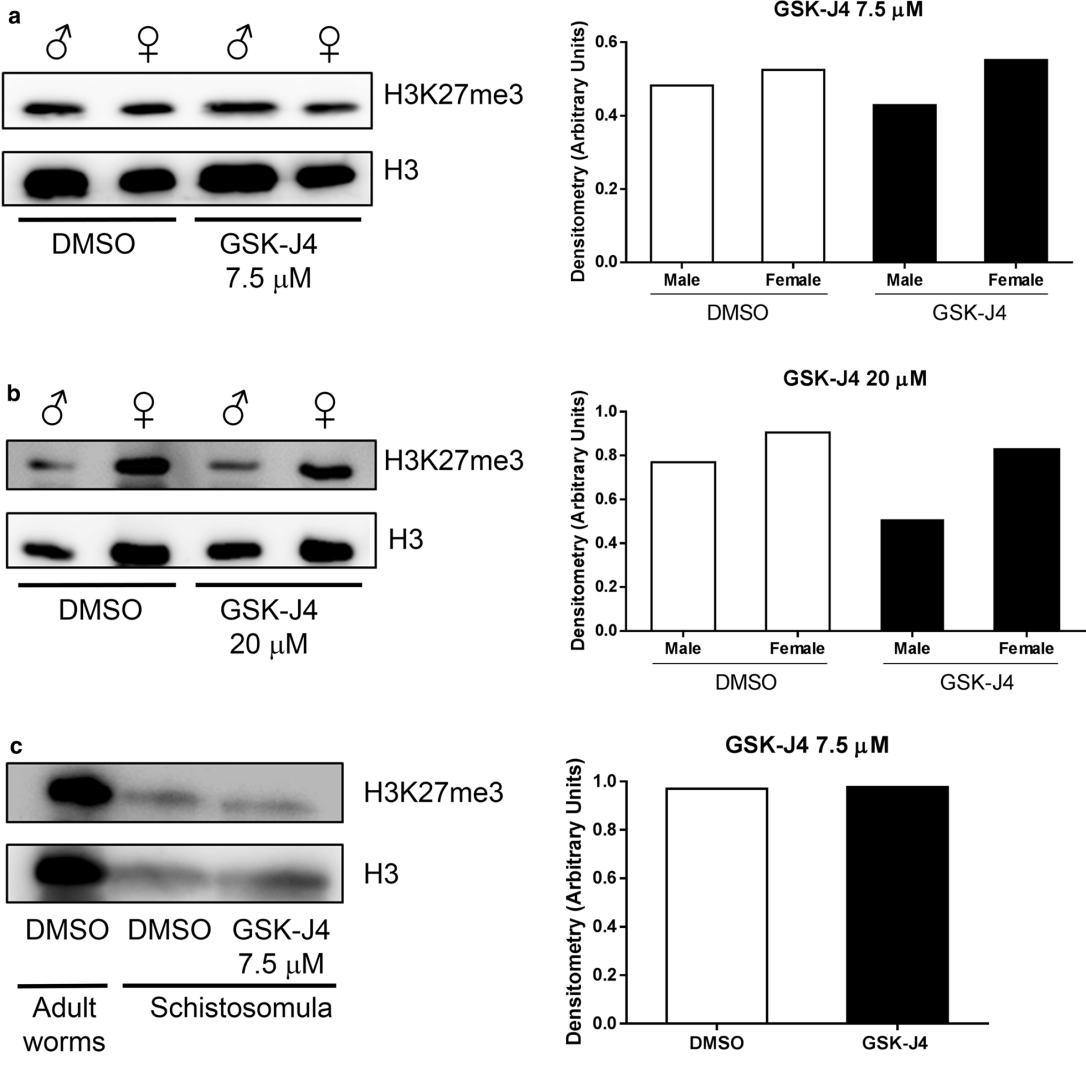
Western blot detection of anti-H3K27me3. **a**, **b** Histones of male and female parasites treated with vehicle (0.1% DMSO), 7.5 µM or 20 µM GSK-J4 for 24 h. **c** Nuclear protein extract of schistosomula treated with vehicle (0.1% DMSO) or with 7.5 μM GSK-J4 for 72 h were submitted to western blotting. Protein extracts were separated by 15% SDS-PAGE, transferred onto a PVDF membrane and submitted to western blotting using monoclonal anti-H3K27me3 (mAbcam 6002) at 1:1000 and anti-H3 core histone (mAbcam 24834) at 1:1000. Densitometry of autoradiograms was performed by calculating the normalized ratio of H3K27me3/H3 core histone signals. Autoradiogram bands were measured by densitometric analysis using ImageJ software (http://rsweb.nih.gov/ij/). Data are representative from one assay

It has been well-established that human KDM6A is associated with gene activation through the demethylation of H3K27me3. To test our working hypothesis that the compound was killing schistosome through the inhibition of Smp_034000, which could interfere with gene expression during the life-cycle, we evaluated the overall methylation levels of H3K27me3 in adult worms and in 3 h schistosomula cultivated for 24 h or 48 h in the presence of the GSK-J4 inhibitor. Surprisingly, western blot analysis demonstrated no increases in the H3K27me3 mark in male or female worms after treatment with 7.5 µM GSK-J4 (Fig. 3a). Moreover, treatment with 20 µM GSK-J4 revealed a slight decrease in H3K27me3 mark levels in adult male worms (Fig. 3b). With respect to schistosomula, incubation with 7.5 µM for 72 h did not result in any alterations in the H3K27me3 mark (Fig. 3c).

### GSK-J4 induces muscle fibers alterations

**Fig. 4:**
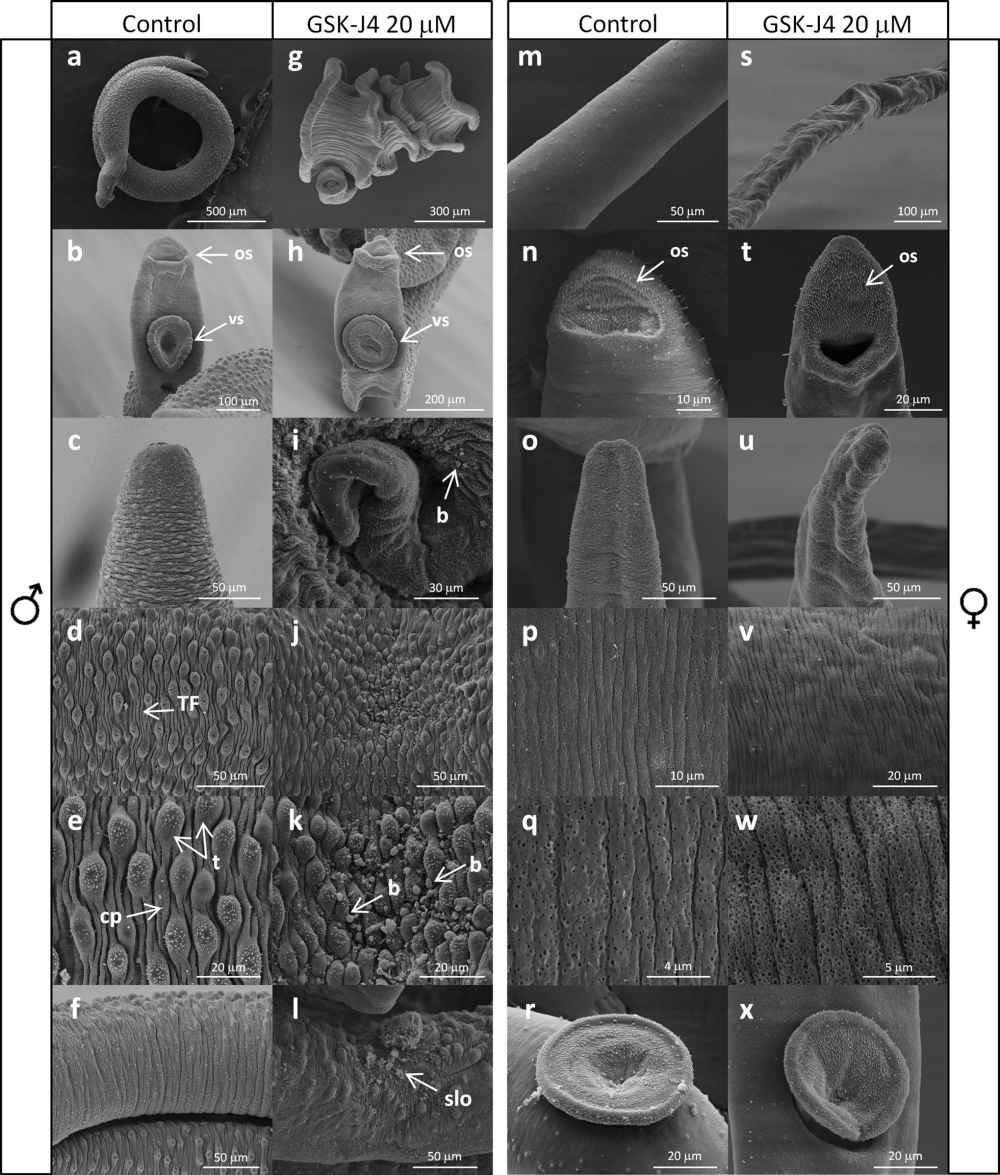
Ultrastructural changes on the body structure of *S. mansoni* adult worms exposed for 24 h to 20 µM GSK-J4. Scanning electron microscopy of control adult male worms (**a**–**e**) and control adult female (**k**–**o**) worms incubated with 0.2% DMSO. Adult male (**f**–**j**) and adult female worms (**p**–**t**) treated with 20 µM GSK-J4. *Abbreviations*: b, blisters; cp, ciliated papillae; os, oral sucker; slo, sloughing; TF, tegumental parallel-arranged folds; t, tubercles; vs, ventral sucker

To gain insights into the possible mechanisms involved in schistosome death, scanning electron and confocal microscopy were used to observe *S. mansoni* adult worms in response to exposure to different concentrations of GSK-J4 compared to controls (Fig. 4a–f, m–r). Scanning electron microscopy demonstrated that males became coiled like corkscrews (Fig. 4g, i), suggesting that their muscle fibers were somehow affected. Overall, the superficial morphology of the tegument seemed similar in control and treated worms (Fig. 4b, h). However, Fig. 4j and k show a downfold of the tegument with a deposit of material that may represent cytoplasm leaking from a damaged area of the tegument surface. This same feature was observed in other surface regions of treated males at varying magnifications (e.g. posterior region, Fig. 4i). Regions near to the gynecophoral canal were severely damaged, presenting sloughing and erosion (Fig. 4l).

The effect of the compound in female worms was more evident, with parasites appearing more elongated with a collapsed body shape (Fig. 4s, u). Regarding the surfaces of females, a greater density in tegument surface pitting was observed in treated females (Fig. 4v, w) (without any signs of cytoplasm leakage), suggesting that tegument function may have been altered. The ventral sucker does not present prominent alterations (Fig. 4r, x).

Next, to investigate internal structures and muscle fiber integrity after compound exposure, Langeron’s carmine and phalloidin-FITC staining was performed. In females, carmine staining revealed that the parenchyma seemed to lose its normal cellular structure, with empty spaces present (Additional file 6: Figure S5), and the cells lining the gut seemed to have lost their normal morphology (Additional file 6: Figure S5d–f), these phenotypic alterations appear to be concentration dependent and were absent in control worms (Additional file 6: Figure S5a) or in worms treated with GSK-J5 (Additional file 6: Figure S5b, c). The worm pairs cultivated in the presence of GSK-J4 laid significantly fewer eggs; thus, we attempted to address by confocal microscopy the effect of the compound in female and male gonads.

Concerning male reproductive system, upon inspection no apparent negative development phenotype was observed; control or treated (GSK-J4 or GSK-J5) male worms present fully developed testicular lobes containing plenty of germinal cells (Additional file 6: Figure S5g–m). However, GSK-J4-treated males revealed some cellular disorganization (cd), evidenced by the presence of some spacing between germinal cells (Additional file 6: Figure S5k–m). Additionally, muscle fibers (mf) were somehow affected at higher concentrations of GSK-J4 (Additional file 6: Figure S5n–s).

Analysis of ovaries from control or GSK-J5 -treated females revealed the typical morphology of a healthy ovary (ov), with a large number of immature and mature oocytes (Additional file 7: Figure S6a–d). In contrast, GSK-J4-treated females revealed some cellular disorganization as evidenced by the presence of some spacing between oocytes, which became more pronounced at 20 μM GSK-J4 (Additional file 7: Figure S6e–g). Further analysis revealed the presence of typical normal eggs inside the ootype (ot) of control female worms with a zygote (z) surrounded by several vitelline cells (vc) and an eggshell with lateral spine (s) (Additional file 7: Figure S6h and n). The ootype derived from females treated with GSK-J5 display maturing eggs (me) with a zygote (z) and some vitelline cells (vc) (Additional file 7: Figure S6i, o, j, p). In ootypes exposed to 800 nM GSK-J4 we observed only a few vitelline cells inside the ootype (ot) with no zygote (Additional file 7: Figure S6k, q); moreover, at high concentrations 7.5 μM and 20 μM GSK-J4 dead eggs with formed eggshells were observed inside the ootype of females (Additional file 7: Figure S6l, r, m, s).

Finally, phalloidin-FITC staining demonstrated that the muscle fibers lost their original features after GSK-J4 exposure at a concentration of 7.5 µM, as muscle fiber staining was very faint (Additional file 8: Figure S7). This was documented through the reapplication of the control parameters (e.g. laser potency, pinhole, gain) (Additional file 8: Figure S7a–d) to capture images of anterior and dorsal regions of treated females (Additional file 8: Figure S7e, f) and male worms (Additional file 8: Figure S7g, h).

## Discussion

Several studies have shown that since *S. mansoni* is strongly dependent on epigenetic mechanisms to control gene expression during development across the parasite life-cycle, epi-drugs could represent a new class of candidates for schistosomiasis treatment [10, 20, 23]. Although the study of anti-schistosomal histone methylation/demethylation inhibitors is still in its infancy, recent investigation on inhibitors of SmEZH2 methyltransferase and Lysine Specific Demethylase 1 (SmLSD1, Smp_150560) have demonstrated the potential of these therapeutic targets [25, 40].

Herein we performed *in silico* screening of the druggability of 14 *Schistosoma* putative histone demethylases, which led us to narrow down our investigation to focus on Smp_034000 using molecular modeling/docking studies and the GSKJ4 inhibitor. Our reanalysis of available transcriptome data indicated that Smp_034000 is highly expressed in 5-week-old adult worms, with male testes being a putative target tissue for exhibiting high expression. Contrary to humans, the presence of only one enzyme isoform (Smp_034000) responsible for H3K27 demethylation in *Schistosoma* suggests that this enzyme may represent a particularly sensitive drug target. *In vitro* treatment with GSK-J4 demonstrated a striking effect in adult worms as well as on schistosomula viability, with mortality observed in a dose- and time-dependent manner.

Recently, 37 epigenetic inhibitors/chemical probes were screened against schistosomula using a high-throughput platform. Similar to our results, GSK-J4 was found to be particularly active against schistosomula and adult worms. In the present study, it was possible to roughly estimate, based on our motility data, that at 72 h the IC_50_ for adult worm motility should be in the range of 5–10 μM, which stands in agreement with the data reported by Whatley et al. [27] (GSK-J4 effect on adult worms motility IC_50_ of 8.97 μM in females and 2.62 μM in males).

However, our results show that, contrary to Whatley et al. [27], the female worms were more severely and rapidly affected by the drug action, as evidenced by a 40% rate of dead females *vs* 0% in males after 72 h of treatment with 10 μM GSK-J4. This finding could be correlated with the more pronounced effect on *in vitro* laid eggs described here and by Whatley et al. [27], revealing that egg production was already reduced at 100 nM GSK-J4. Effect of the compound on adult female worms became more evident by scanning electron microscopy, as parasites appeared more elongated, with a collapsed body shape and increased pitting. Cellular disorganization, with cells appearing loose, was observed in different internal tissues (e.g. parenchyma, ovaries and testes). This effect seems to affect all tissue with no evidence of a propensity to affect the gonads. These data, together with confocal microcopy results following phalloidin-FITC staining, strongly suggests that GSK-J4 may affect the integrity of cellular junctions (packing) and muscle fibers and this seems to be more evident on adult female worms.

Our data did indicate that the inactive permeable isomer (GSK-J5) had no major effect on motility and mortality of adult worms, but did inhibit egg-laying, similarly to GSK-J4, at a concentration of 30 μM. Contrary to Whatley et al. [27], we observed significant egg size reduction (~18%) after treatment with 200 nM GSK-J4, which became more evident (~66%) with 800 nM GSK-J4. Unexpectedly, 20 μM GSK-J5 did reduce egg size (~20%); however, the phenotypic alterations seem to be different from that observed for GSK-J4 treatment.

The egg formation is a very complex process with two pre-embryonic stages occurring inside the female worm. The prezygotic stage, which is characterized by the release of mature oocytes from the female ovary. Followed by the zygotic stage that includes the migration of the zygote through the ootype, where the eggshell is formed, to finally pass the uterus and be released through the gonopore. In the outside environment eggs increase significantly in size during the development of eight embryonic stages [44, 45]. We believe that the reduction in size observed after GSK-J4 exposure occurs during egg formation inside the female worm and not after its release into the culture medium. Accordingly, we have tried to address this issue by analyzing the ootype of paired females by confocal laser scanning microscopy. Our preliminary findings suggested that the compound could affect the packing of vitteline cells and the zygote inside the ootype, but additional analyses must be carried out to reach statistically significant data. This is in agreement with results of Whatley et al. [27], who described that 200 nM GSK-J4 inhibits packaging of vitelline cells in laid eggs. Although our study did not assess the packing of vitelline cells in laid eggs, our data indicate similar phenotypic egg alterations after exposure to 800 nM GSK-J4.

Schistosomula, which have demonstrated resistance to praziquantel [43], present a GSK-J4 IC_50_ of 4.2 μM based on the ATP reduction, which is similar to that described by Whatley et al. [27] (IC_50_ of 5.3–6.0 μM). Microscopic assessment of schistosomula treated with 6.25 μM demonstrated a granulation phenotype similar to that observed by Whatley et al. [27]. Overall, the strong correlations between the present data and those described by Whatley et al. [27] stand in agreement with previous findings in the literature stating that high throughput screening correctly detected 99.8% of the visually or semi-automatic scored hits [46]. Based on our domain architecture analysis, considering that Smp_034000 is more closely related to human KDM6A (UTX) than to human KDM6B (JmJD3), we, as well as other authors [23, 47], propose that it is more appropriate to classify Smp_034000 as a *Schistosoma* KDM6A/UTX.

Our attempt to initially validate the inhibition of Smp_034000 by GSK-J4 *via* western blotting was unable to confirm the modulation of H3K27me3. One possibility for this could be akin to what was recently observed in the treatment of different prostate cancer cell lines with GSK-J4. Morozov et al. [48] similarly found no modulation of H3K27me3 but did observe a decrease in H3K27me1 levels. These authors argue that the mechanistic interpretation of this phenomenon is that the inhibition of JMJD3/UTX by GSK-J4 did not induce a global accumulation of H3K27me3, but rather elevated the levels of this mark in specific promoter regions [48]. Thus, the question remains: is this indeed the case in the schistosome?

Another possibility could be related to initial findings describing GSK-J4 as selectively inhibiting two human demethylases (KDM6A and KDM6B), which share conserved domains and activities [26]. However, Heinemann et al. [49] later demonstrated that GSK-J4 also inhibits KDM5B and KDM5D, albeit to a lesser extent. It is important to note that since orthologs of these two demethylases are predicted in *S. mansoni*, we cannot rule out the possibility that GSK-J4 may also be acting by way of other mechanisms.

## Conclusions

Despite these unresolved questions, the involvement of GSK-J4 on Smp_34000 inhibition warrants further investigation. RNAi assays against Smp_34000 could revisit the GSK-J4 drug effect phenotype to provide evidence that Smp_34000 is, in fact, an active KDM6A/UTX lysine demethylase required for schistosomula transformation, egg production and H3K27 demethylation. Another possibility would be the use of chemoproteomics to reliably determine whether Smp_34000 interacts with GSK-J4, as performed by Kruidenier et al. [26] for the human ortholog. Upon achieving this goal, schistosome Smp_034000 protein demethylase should undergo validation as an anthelmintic target.

## Supplementary information

**Additional file 1: Figure S1.** Comparison of *S. mansoni* demethylase domain architecture and corresponding human orthologs. Proteins domains were mapped using SMART. This analysis includes only *S. mansoni* proteins presenting druggability score > 0.8.

**Additional file 2: Figure S2.** Smp_034000 transcription profile across the parasite life-cycle and gonad-specific and pairing-dependent study.

**Additional file 3: Figure S3.** Comparison between the catalytic domain of the *Schistosoma* UTX protein sequence and human UTX/KDM6A, UTY and JMJD3/KDM6B. Alignments were generated by ClustalX. Residues involved in histone binding (red stars) and residues participating in cofactor (NOG) and metal binding (blue circles) are highlighted. Green triangles indicate cysteines participating in Zinc binding. Regions with high identity and similarity between protein sequences are shown as black and gray columns, according to the Clustal X algorithm.

**Additional file 4: Figure S4.** Decrease in egg size and oviposition in *S. mansoni* couples exposed to GSK-J4 and GSK-J5 for 72 h. Data are expressed as mean ± SEM from one experiment, data for 30 µM GSK-J5 point was replotted from the previous assay (Fig. 2c) for comparison. *P < 0.05, **P < 0.01 and ***P < 0.001.

**Additional file 5: Movie S1.** Microscopic assessment of schistosomula after 48 h of treatment with GSK-J4 6.25 μM, demonstrating impaired motility and mortality with a granulation phenotype.

**Additional file 6: Figure S5.** Confocal micrographs of *Schistosoma mansoni* adult worms exposed to GSK-J4 or GSK-J5.

**Additional file 7: Figure S6.** Confocal micrographs of the reproductive organs of *Schistosoma mansoni* female adult worms exposed to GSK-J4 or GSK-J5. Panel **a** was  adapted from [50].

**Additional file 8: Figure S7.** Confocal micrographs of muscle fibers of *Schistosoma mansoni* adult worms exposed to GSK-J4 7.5 μM for 24 h. Male and female worms stained with phalloidin-FITC revealing that muscle fibers lose their original features after GSK-J4 exposure.

## Data Availability

Data supporting the conclusions of this article are included within the article and its additional files. The datasets generated and/or analyzed during the current study are available in the ArrayExpress repository, (https://www.ebi.ac.uk/arrayexpress/experiments/E-MEXP-2094/) and in the European Nucleotide Archive repository (https://www.ebi.ac.uk/ena/browser/view/PRJEB14695).

## Abbreviations

PZQ: praziquantel
NTD: neglected tropical disease
HDAC: histone deacetylase
HAT: histone acetyltransferase
GPCRs: G protein-coupled receptors
HMT: histone methyltransferase
H3K9ac: acetylation of histone H3 at lysine 9
H3K27me3: trimethylation of histone H3 at lysine 27
HDM: histone demethylase
KDM: K-demethylase
LSD: lysine specific demethylase
JMJC: Jumonji C
SmHDM: *S. mansoni* histone demethylases
JMJD3: Jumonji domain-containing protein 3
KDM6A: lysine demethylase 6A
UTX: ubiquitously transcribed tetratricopeptide repeat X chromosome
UTY: ubiquitously transcribed tetratricopeptide repeat Y chromosome
SMART: Simple Modular Architecture Research Tool
DMSO: dimethyl sulfoxide
TCA: trichloroacetic acid
DIC: differential interference contrast microscopy
FITC: fluorescein isothyocianate
DAPI: 4′,6-diamidin-2-phenylindol
