# Supplementary material for: The antischistosomal potential of GSK-J4, an H3K27 demethylase inhibitor: insights from molecular modeling, transcriptomics and in vitro assays
Source: Parasit Vectors. 2020 Mar 17;13:140. doi: 10.1186/s13071-020-4000-z (PMC7077139; doi:10.1186/s13071-020-4000-z)
Supplement: Supplementary file 1 — Additional file 1: Figure S1. Comparison of S. mansoni demethylase domain architecture and corresponding human orthologs. Proteins domains were mapped using SMART. This analysis includes only S. mansoni proteins presenting druggability score > 0.8. [file 13071_2020_4000_MOESM1_ESM.pptx]

## Slide 1
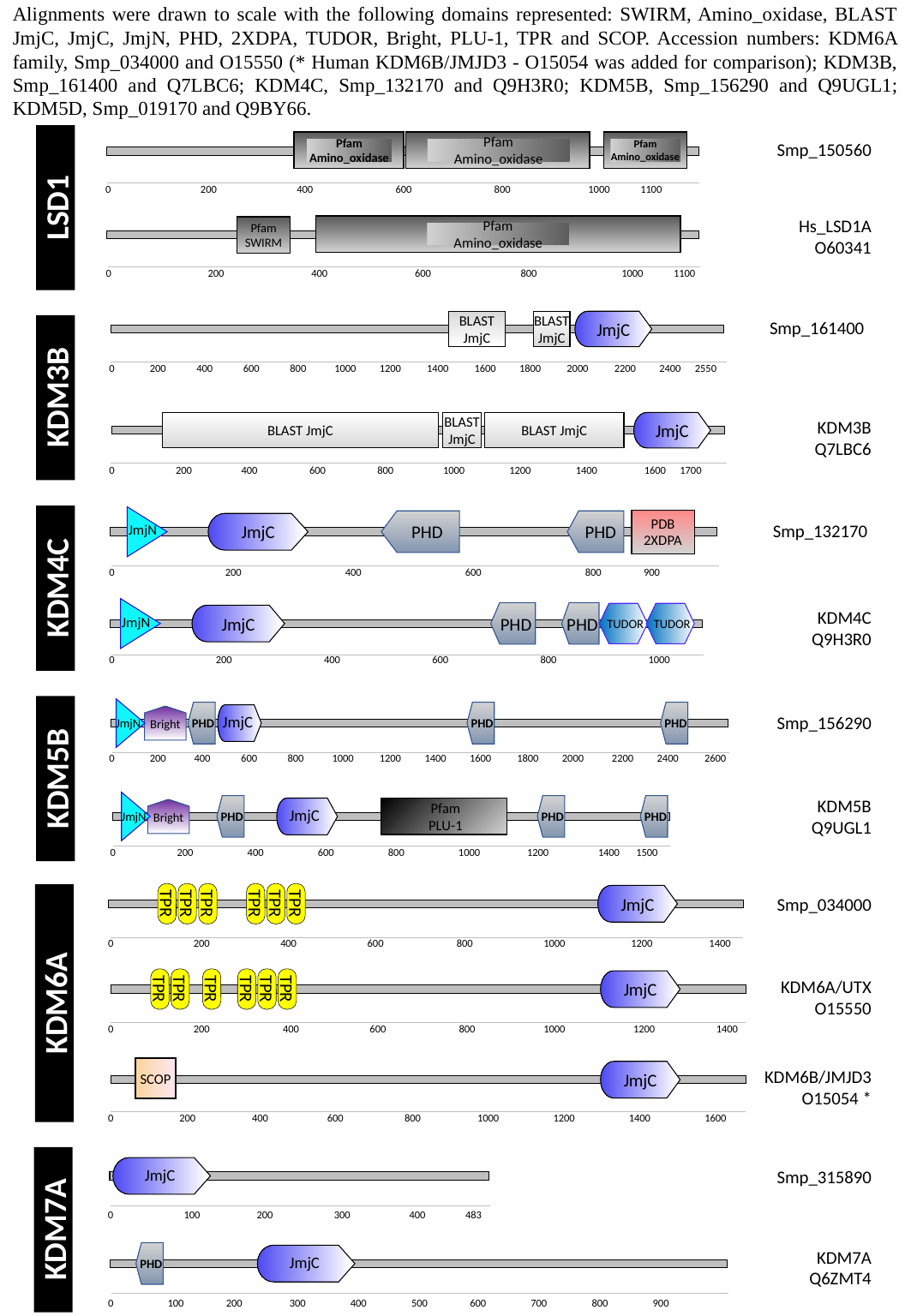

Alignments were drawn to scale with the following domains represented: SWIRM, Amino_oxidase, BLAST JmjC, JmjC, JmjN, PHD, 2XDPA, TUDOR, Bright, PLU-1, TPR and SCOP. Accession numbers: KDM6A family, Smp_034000 and O15550 (* Human KDM6B/JMJD3 - O15054 was added for comparison); KDM3B, Smp_161400 and Q7LBC6; KDM4C, Smp_132170 and Q9H3R0; KDM5B, Smp_156290 and Q9UGL1; KDM5D, Smp_019170 and Q9BY66.
Pfam
Amino_oxidase
Pfam
Amino_oxidase
Pfam
Amino_oxidase
0 200 400 600 800 1000 1100
Smp_150560
LSD1
Hs_LSD1A
O60341
Pfam
Amino_oxidase
Pfam SWIRM
0 200 400 600 800 1000 1100
Smp_161400
JmjC
BLAST JmjC
BLAST JmjC
0 200 400 600 800 1000 1200 1400 1600 1800 2000 2200 2400 2550
KDM3B
JmjC
BLAST JmjC
BLAST JmjC
BLAST JmjC
0 200 400 600 800 1000 1200 1400 1600 1700
KDM3B
Q7LBC6
JmjN
PDB 2XDPA
PHD
PHD
JmjC
0 200 400 600 800 900
 Smp_132170
KDM4C
JmjN
PHD
TUDOR
TUDOR
JmjC
PHD
0 200 400 600 800 1000
KDM4C
Q9H3R0
JmjN
Bright
PHD
JmjC
PHD
PHD
0 200 400 600 800 1000 1200 1400 1600 1800 2000 2200 2400 2600
Smp_156290
KDM5B
JmjN
Bright
PHD
PHD
PHD
JmjC
Pfam
PLU-1
0 200 400 600 800 1000 1200 1400 1500
KDM5B
Q9UGL1
JmjC
TPR
TPR
TPR
TPR
TPR
TPR
0 200 400 600 800 1000 1200 1400
Smp_034000
JmjC
TPR
TPR
TPR
TPR
TPR
TPR
0 200 400 600 800 1000 1200 1400
KDM6A/UTX
 O15550
KDM6A
SCOP
JmjC
0 200 400 600 800 1000 1200 1400 1600
KDM6B/JMJD3
 O15054 *
Smp_315890
JmjC
0 100 200 300 400 483
KDM7A
KDM7A
 Q6ZMT4
PHD
JmjC
0 100 200 300 400 500 600 700 800 900
