# Supplementary material for: The antischistosomal potential of GSK-J4, an H3K27 demethylase inhibitor: insights from molecular modeling, transcriptomics and in vitro assays
Source: Parasit Vectors. 2020 Mar 17;13:140. doi: 10.1186/s13071-020-4000-z (PMC7077139; doi:10.1186/s13071-020-4000-z)
Supplement: Supplementary file 2 — Additional file 2: Figure S2. Smp_034000 transcription profile across the parasite life-cycle and gonad-specific and pairing-dependent study. [file 13071_2020_4000_MOESM2_ESM.pptx]

## Slide 1
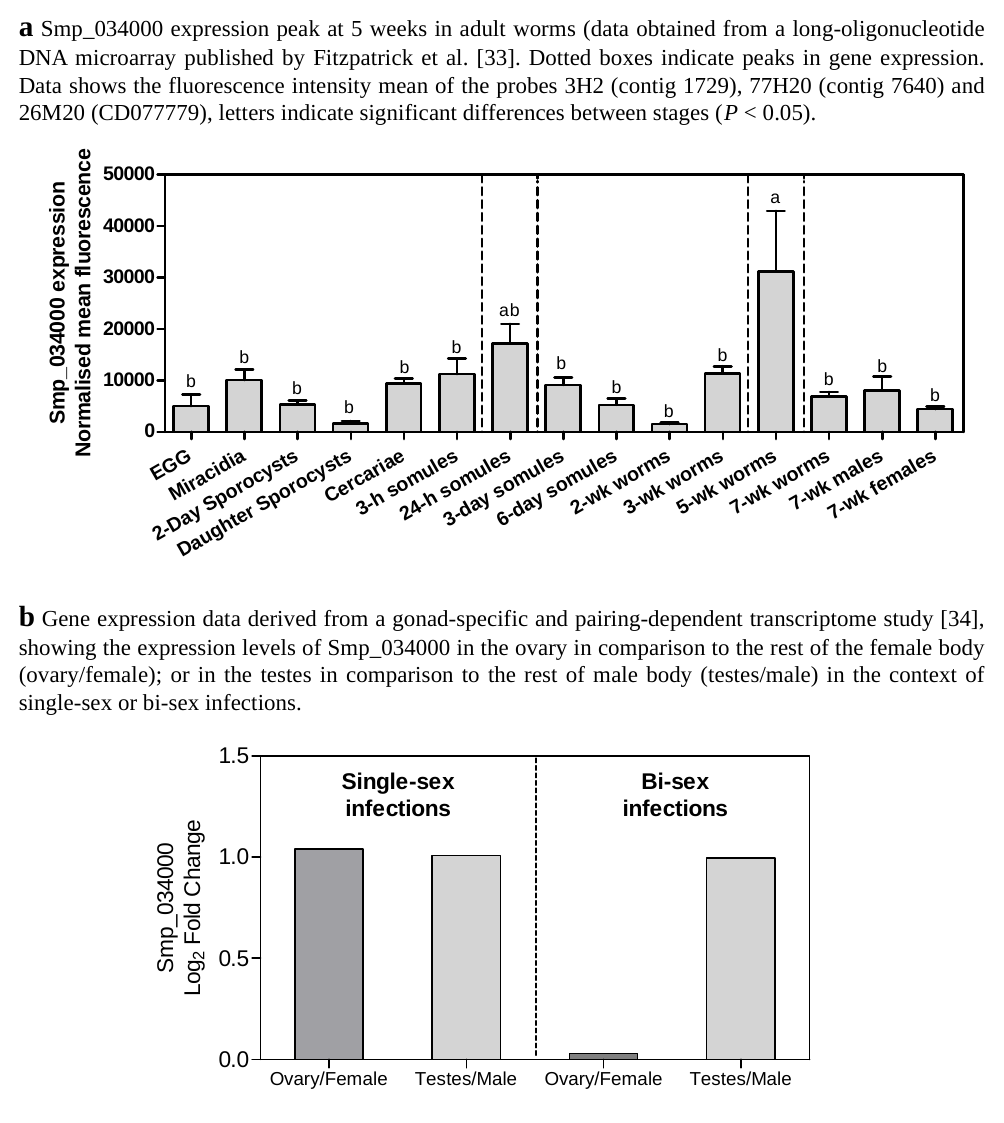

a Smp_034000 expression peak at 5 weeks in adult worms (data obtained from a long-oligonucleotide DNA microarray published by Fitzpatrick et al. [33]. Dotted boxes indicate peaks in gene expression. Data shows the fluorescence intensity mean of the probes 3H2 (contig 1729), 77H20 (contig 7640) and 26M20 (CD077779), letters indicate significant differences between stages (P < 0.05).
b Gene expression data derived from a gonad-specific and pairing-dependent transcriptome study [34], showing the expression levels of Smp_034000 in the ovary in comparison to the rest of the female body (ovary/female); or in the testes in comparison to the rest of male body (testes/male) in the context of single-sex or bi-sex infections.
