# Supplementary material for: The antischistosomal potential of GSK-J4, an H3K27 demethylase inhibitor: insights from molecular modeling, transcriptomics and in vitro assays
Source: Parasit Vectors. 2020 Mar 17;13:140. doi: 10.1186/s13071-020-4000-z (PMC7077139; doi:10.1186/s13071-020-4000-z)
Supplement: Supplementary file 3 — Additional file 3: Figure S3. Comparison between the catalytic domain of the Schistosoma UTX protein sequence and human UTX/KDM6A, UTY and JMJD3/KDM6B. Alignments were generated by ClustalX. Residues involved in histone binding (red stars) and residues participating in cofactor (NOG) and metal binding (blue circles) are highlighted. Green triangles indicate cysteines participating in Zinc binding. Regions with high identity and similarity between protein sequences are shown as black and gray columns, according to the Clustal X algorithm. [file 13071_2020_4000_MOESM3_ESM.pdf]

```
                          1160                1180                1200
UTX Human    AEVLKACRNLGKNGLSNSSILLDKCPPPRPPSSPYPPLPKDKLNPPTPSIYLENKRDAFFPPLHQFC  960
UTY Human    TEVLKACRNPGKNGLSNSCILLDKCPPPRPPTSPYPPLPKDKLNPPTPSIYLENKRDAFFPPLHQFC  907
Smp 034000   ---LRGLGHSG--GPWWPGLLPEGSPIPKPPAKPYPPPPKDKLLPPTPSVYLENRNDAHSPELMRYC  640
JMJD3 Human  ADVVRASRNAKVKGKFRESYLSP-AQSVKPKINTEEKLPREKLNPPTPSIYLESKRDAFSPVLLQFC 1205

                    1220                1240                1260
UTX Human    TNPNNPVTVIRGLAGALKLDLGLFSTKTLVEANNEHMVEVRTQLLQPADENWDPTGTKKIWHCESNR 1027
UTY Human    TNPKNPVTVIRGLAGALKLDLGLFSTKTLVEANNEHMVEVRTQLLQPADENWDPTGTKKIWRCESNR  974
Smp 034000   FT--QPVVVIRGLAAALRLDLGLFSTKSLVESNPEHRVEVRTQRLQSPDQNLDSQG-RTVWLCESPK  704
JMJD3 Human  TDPRNPITVIRGLAGSLRLNLGLFSTKTLVEASGEHTVEVRTQVQQPSDENWDLTGTRQIWPCESSR 1272

               1280                1300                1320                1340
UTX Human    SHTTIAKYAQYQASSFQESLREE---------------------------NEKRS-----------H 1056
UTY Human    SHTTIAKYAQYQASSFQESLREE---------------------------NEKRT-----------Q 1003
Smp 034000   TTTTIAKYGAYQAASFIEAMKEEKIVNFSGSAPVTSTPTSNTISAISVDTKSSTGNLSNAVTGSGRK  771
JMJD3 Human  SHTTIAKYAQYQASSFQESLQEE---------------------------KES-------------E 1299

                          1360                1380                1400
UTX Human    HKDHSDSESTSSDN--------------------SGRRRKGPFKTIKFGTNIDLSDDKKWKLQLHEL 1103
UTY Human    HKDHSDNESTSSEN--------------------SGRRRKGPFKTIKFGTNIDLSDNKKWKLQLHEL 1050
Smp 034000   NSTHWSSESTNSELNVNQPHPVGSKLSGDENNSLSNQQNSGRLKLIRFGTNCDLSDQKKWLPQLHEL  838
JMJD3 Human  DEESEEPDSTTGTPP-------------------SSAPDPKNHHIIKFGTNIDLSDAKRWKPQLQEL 1347

                    1420                1440                1460
UTX Human    TKLPAFVRVVSAGNLLSHVGHTILGMNTVQLYMKVPGSRTPGHQENNNFCSVNINIGPGDCEWFVVP 1170
UTY Human    TKLPAFARVVSAGNLLTHVGHTILGMNTVQLYMKVPGSRTPGHQENNNFCSVNINIGPGDCEWFVVP 1117
Smp 034000   TKLPIFVRVVSAFNMLSHVGYPLLGLNTVQLYLKVPGSRTPGHQENNNFCAVNINIGPGDCEWFSVP  905
JMJD3 Human  LKLPAFMRVTSTGNMLSHVGHTILGMNTVQLYMKVPGSRTPGHQENNNFCSVNINIGPGDCEWFAVH 1414

                1480                1500                1520                1540
UTX Human    EGYWGVLNDFCEKNNLNFLMGSWWPNLEDLYEANVPVYRFIQRPGDLVWINAGTVHWVQAIGWCNNI 1237
UTY Human    EDYWGVLNDFCEKNNLNFLMSSWWPNLEDLYEANVPVYRFIQRPGDLVWINAGTVHWVQAVGWCNNI 1184
Smp 034000   EQYWGAIHNLCEKNNVDYLTGSWWPDLETLYKEEIPVYRFIQRPGDLVWINAGTVHWVQAIGWCNNI  972
JMJD3 Human  EHYWETISAFCDRHGVDYLTGSWWPILDDLYASNIPVYRFVQRPGDLVWINAGTVHWVQATGWCNNI 1481

                         1560                1580                1600
UTX Human    AWNVGPLTACQYKLAVERYEWNKLQSVKSIVPMVHLSWNMARNIKVSDPKLFEMIKYCLLRTLKQCQ 1304
UTY Human    AWNVGPLTACQYKLAVERYEWNKLKSVKSPVPMVHLSWNMARNIKVSDPKLFEMIKYCLLKILKQYQ 1251
Smp 034000   AWNVGCMTARQYQLAVERYEFNRLRGVKSVVPMTHLSWQLAKNIKISDPGLFELIKHTLLRSFIQSQ 1039
JMJD3 Human  AWNVGPLTAYQYQLALERYEWNEVKNVKSIVPMIHVSWNVARTVKISDPDLFKMIKFCLLQSMKHCQ 1548

                   1620                1640                1660
UTX Human    TLREALIAAGKEIIWHGRTKEEPAHYCSICEVEVFDLLFVTNESNSRKTYIVHCQDCARKTSGNLEN 1371
UTY Human    TLREALVAAGKEVIWHGRTNDEPAHYCSICEVEVFNLLFVTNESNTQKTYIVHCHDCARKTSKSLEN 1318
Smp 034000   LTTDFLEKMNLTIKHHGKRSDDIAHSCHDCEIEVFNILFVLSQD---KKLVVRCLDCARRMDSTLKT 1103
JMJD3 Human  VQRESLVRAGKKIAYQGRVKDEPAYYCNECDVEVFNILFVTSENGSRNTYLVHCEGCARRRSAGLQG 1615

               1680                1700
UTX Human    FVVLEQYKMEDLMQVYDQFTLAPPLPSASS---- 1401
UTY Human    FVVLEQYKMEDLIQVYDQFTLALSLSSSS----- 1347
Smp 034000   FIILSEYHIHELAEIFDKFQLQTQPITAYPVGKT 1137
JMJD3 Human  VVVLEQYRTEELAQAYDAFTLAPASTSR------ 1643
```
