# Supplementary material for: The antischistosomal potential of GSK-J4, an H3K27 demethylase inhibitor: insights from molecular modeling, transcriptomics and in vitro assays
Source: Parasit Vectors. 2020 Mar 17;13:140. doi: 10.1186/s13071-020-4000-z (PMC7077139; doi:10.1186/s13071-020-4000-z)
Supplement: Supplementary file 4 — Additional file 4: Figure S4. Decrease in egg size and oviposition in S. mansoni couples exposed to GSK-J4 and GSK-J5 for 72 h. Data are expressed as mean ± SEM from one experiment, data for 30 µM GSK-J5 point was replotted from the previous assay (Fig. 2c) for comparison. *P < 0.05, **P < 0.01 and ***P < 0.001. [file 13071_2020_4000_MOESM4_ESM.pptx]

## Slide 1
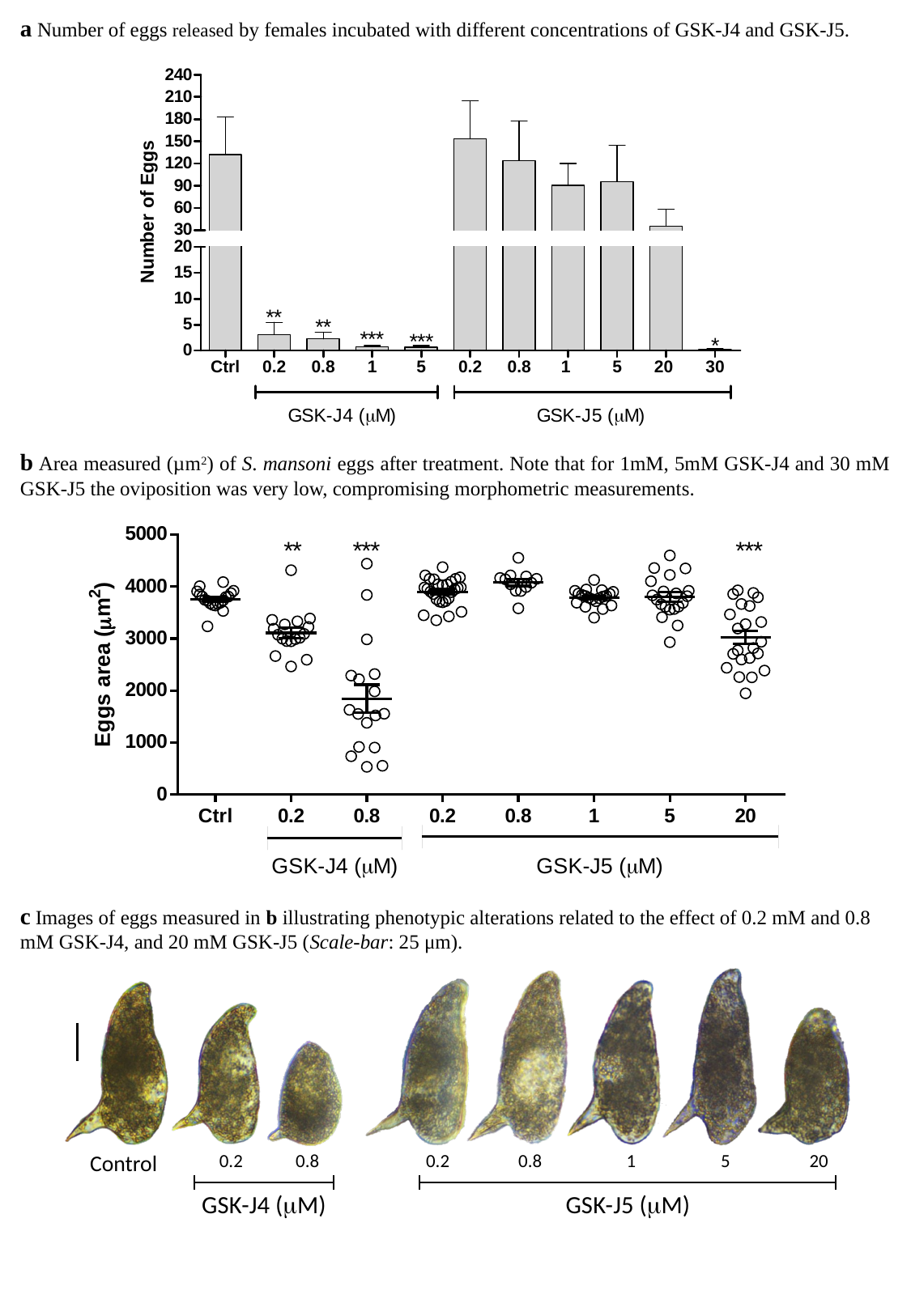

a Number of eggs released by females incubated with different concentrations of GSK-J4 and GSK-J5.
b Area measured (µm2) of S. mansoni eggs after treatment. Note that for 1mM, 5mM GSK-J4 and 30 mM GSK-J5 the oviposition was very low, compromising morphometric measurements.
c Images of eggs measured in b illustrating phenotypic alterations related to the effect of 0.2 mM and 0.8 mM GSK-J4, and 20 mM GSK-J5 (Scale-bar: 25 μm).
Control
0.2
0.8
0.2
0.8
1
5
20
GSK-J4 (μM)
GSK-J5 (μM)
