# Supplementary material for: The antischistosomal potential of GSK-J4, an H3K27 demethylase inhibitor: insights from molecular modeling, transcriptomics and in vitro assays
Source: Parasit Vectors. 2020 Mar 17;13:140. doi: 10.1186/s13071-020-4000-z (PMC7077139; doi:10.1186/s13071-020-4000-z)
Supplement: Supplementary file 6 — Additional file 6: Figure S5. Confocal micrographs of Schistosoma mansoni adult worms exposed to GSK-J4 or GSK-J5. [file 13071_2020_4000_MOESM6_ESM.pptx]

## Slide 1
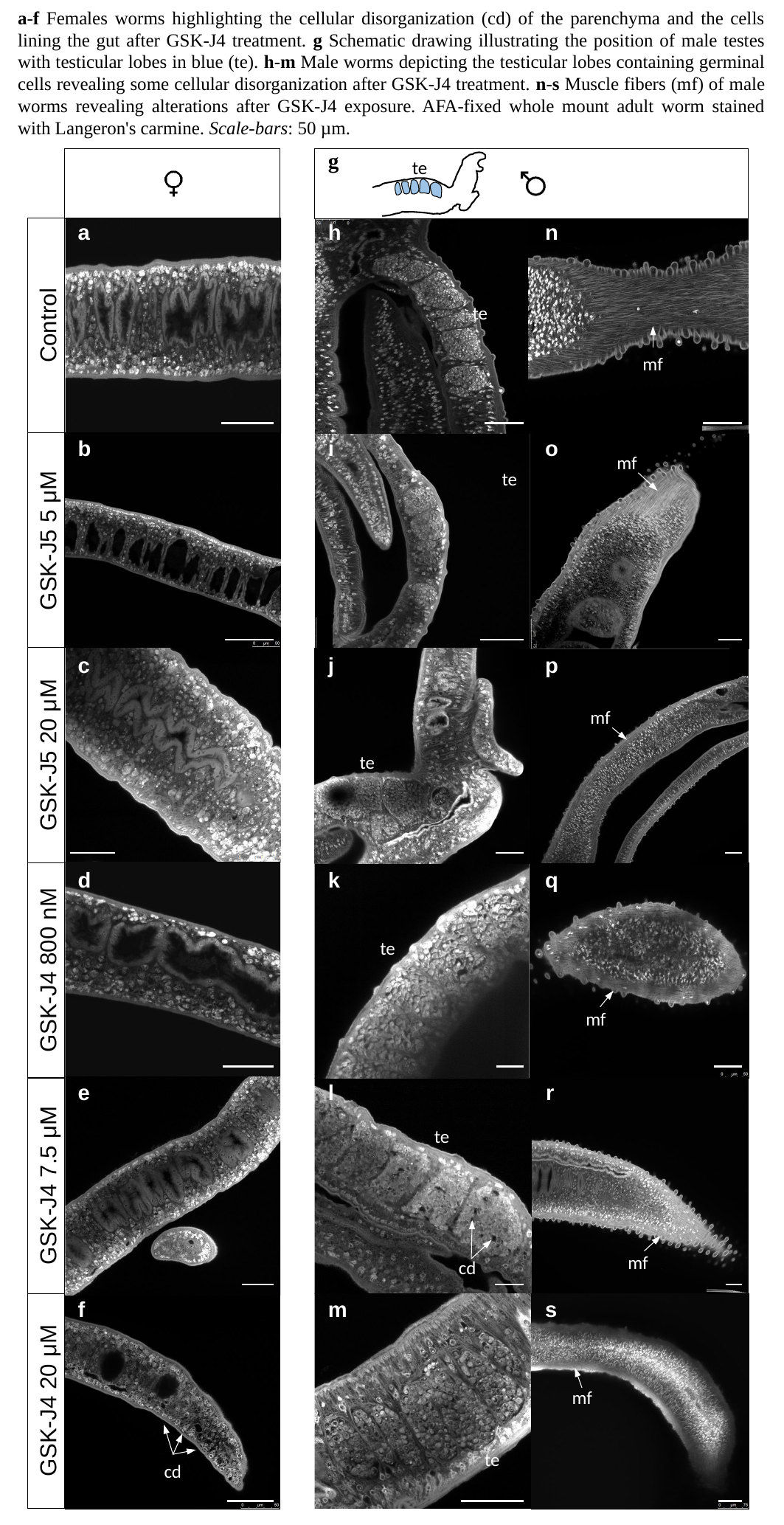

a-f Females worms highlighting the cellular disorganization (cd) of the parenchyma and the cells lining the gut after GSK-J4 treatment. g Schematic drawing illustrating the position of male testes with testicular lobes in blue (te). h-m Male worms depicting the testicular lobes containing germinal cells revealing some cellular disorganization after GSK-J4 treatment. n-s Muscle fibers (mf) of male worms revealing alterations after GSK-J4 exposure. AFA-fixed whole mount adult worm stained with Langeron's carmine. Scale-bars: 50 µm.
g
te
a
h
n
te
Control
mf
b
i
o
mf
te
GSK-J5 5 μM
c
j
p
mf
GSK-J5 20 μM
te
d
k
q
te
GSK-J4 800 nM
mf
e
l
r
te
GSK-J4 7.5 μM
mf
cd
f
m
s
mf
GSK-J4 20 μM
te
cd
