# Supplementary material for: The antischistosomal potential of GSK-J4, an H3K27 demethylase inhibitor: insights from molecular modeling, transcriptomics and in vitro assays
Source: Parasit Vectors. 2020 Mar 17;13:140. doi: 10.1186/s13071-020-4000-z (PMC7077139; doi:10.1186/s13071-020-4000-z)
Supplement: Supplementary file 7 — Additional file 7: Figure S6. Confocal micrographs of the reproductive organs of Schistosoma mansoni female adult worms exposed to GSK-J4 or GSK-J5. Panel a was adapted from [50]. [file 13071_2020_4000_MOESM7_ESM.pptx]

## Slide 1
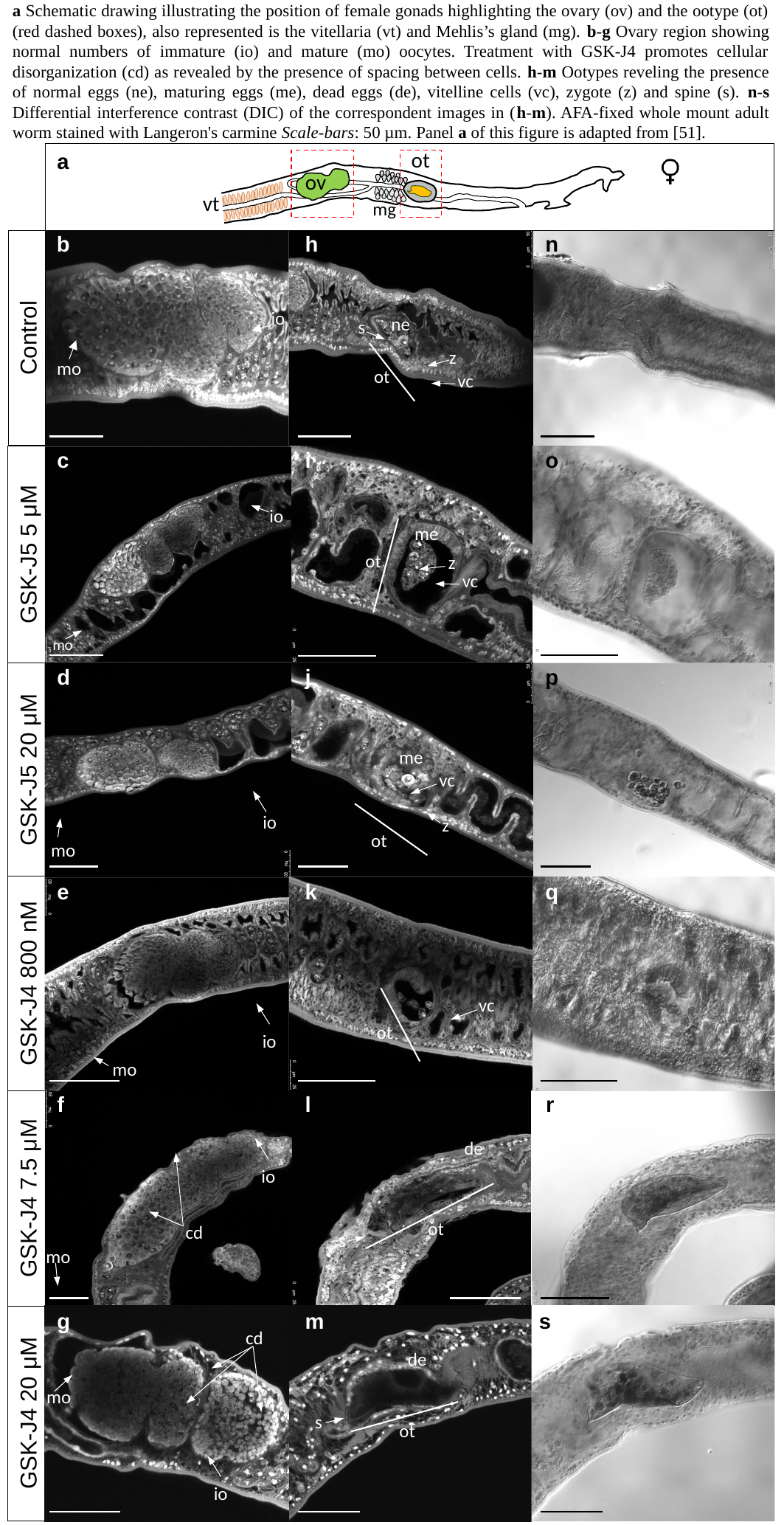

a Schematic drawing illustrating the position of female gonads highlighting the ovary (ov) and the ootype (ot) (red dashed boxes), also represented is the vitellaria (vt) and Mehlis’s gland (mg). b-g Ovary region showing normal numbers of immature (io) and mature (mo) oocytes. Treatment with GSK-J4 promotes cellular disorganization (cd) as revealed by the presence of spacing between cells. h-m Ootypes reveling the presence of normal eggs (ne), maturing eggs (me), dead eggs (de), vitelline cells (vc), zygote (z) and spine (s). n-s Differential interference contrast (DIC) of the correspondent images in (h-m). AFA-fixed whole mount adult worm stained with Langeron's carmine Scale-bars: 50 µm. Panel a of this figure is adapted from [51].
ot
ov
vt
mg
a
b
h
n
io
ne
s
Control
z
mo
ot
vc
c
i
o
io
me
GSK-J5 5 μM
ot
z
vc
mo
d
j
p
me
GSK-J5 20 μM
vc
io
z
ot
mo
e
k
q
GSK-J4 800 nM
vc
ot
io
mo
f
l
r
de
io
GSK-J4 7.5 μM
ot
cd
s
mo
g
m
s
cd
de
mo
GSK-J4 20 μM
s
ot
io
