# Supplementary material for: The antischistosomal potential of GSK-J4, an H3K27 demethylase inhibitor: insights from molecular modeling, transcriptomics and in vitro assays
Source: Parasit Vectors. 2020 Mar 17;13:140. doi: 10.1186/s13071-020-4000-z (PMC7077139; doi:10.1186/s13071-020-4000-z)
Supplement: Supplementary file 8 — Additional file 8: Figure S7. Confocal micrographs of muscle fibers of Schistosoma mansoni adult worms exposed to GSK-J4 7.5 μM for 24 h. Male and female worms stained with phalloidin-FITC revealing that muscle fibers lose their original features after GSK-J4 exposure. [file 13071_2020_4000_MOESM8_ESM.pptx]

## Slide 1
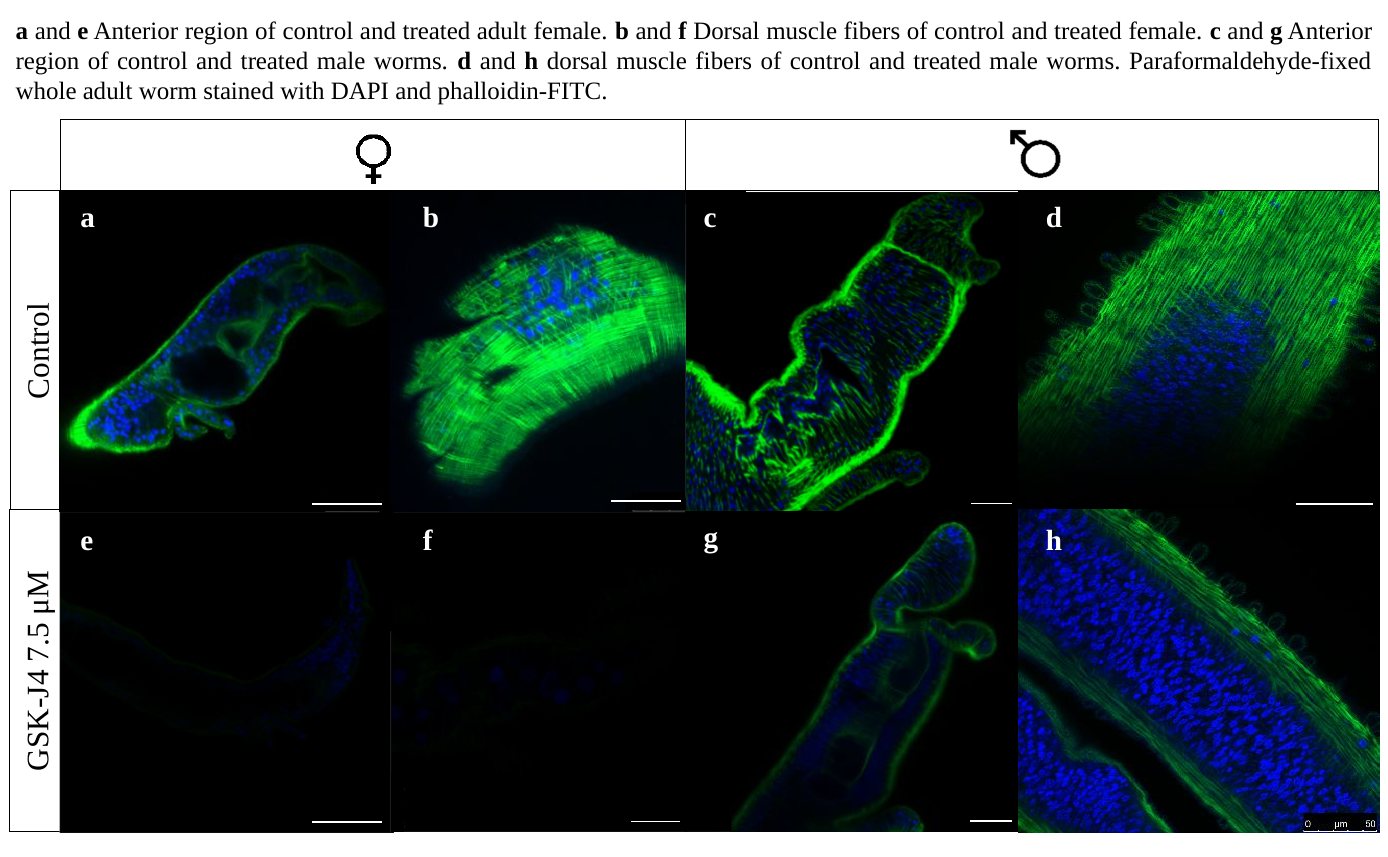

a and e Anterior region of control and treated adult female. b and f Dorsal muscle fibers of control and treated female. c and g Anterior region of control and treated male worms. d and h dorsal muscle fibers of control and treated male worms. Paraformaldehyde-fixed whole adult worm stained with DAPI and phalloidin-FITC.
a
b
c
d
g
e
f
h
Control
GSK-J4 7.5 μM
